# What do cardiac surgical assistants in the Kingdom of Saudi Arabia think about their job? A cross-sectional survey of job and work characteristics

**DOI:** 10.1177/17504589211022593

**Published:** 2021-07-29

**Authors:** Mohammed Bahran Shegafi, Mary Leamy, Trevor Murrells, Geraldine A Lee

**Affiliations:** 1 Kings College London, Florence Nightingale Faculty of Nursing, Midwifery & Palliative Care, London, UK; 2 Cardiac Surgery Department, King Abdullah Medical City, Makkah, Saudi Arabia

**Keywords:** Surgical Assistants, Surgical care practice, Job design, Survey, Organisation

## Abstract

Concerns about job design of the cardiac surgical assistant workforce such as role autonomy and job dissatisfaction have been outlined in the literature, although scant empirical research has examined these concerns from the perspective of cardiac surgical assistants themselves. This study surveyed the job design of cardiac surgical assistants in the Kingdom of Saudi Arabia using Morgeson and Humphrey’s Work Design Questionnaire. All scalable items within the questionnaire were reported as satisfactory except for ‘autonomy’, ‘task identity’, ‘feedback from the job’, ‘job complexity’, ‘social support’, ‘feedback from others’, ‘ergonomic’ and ‘work condition’. The results provide insight into aspects of cardiac surgical assistants’ role characteristics and contribute to the body of knowledge about their organisational psychology. Given the growth of cardiothoracic operations, the role of the surgical care assistant needs to be further developed to address the job design issues raised.

**Provenance and Peer review:** Unsolicited contribution; Peer reviewed; Accepted for publication 16 May 2021.

## Introduction

Coronary Artery Bypass Graft (CABG) surgery is one of the most commonly performed major procedures, with an estimated 200,000 isolated CABG procedures performed globally per annum (Squiers & Mack 2018). There is a global chronic shortage of cardiac surgeons, and the increasing volume of CABG operations has not been matched with an increase in the number of cardiothoracic trainee surgeons (Grover et al 2009, Yan et al 2021). To address this gap, the Kingdom of Saudi Arabia (KSA), like other countries around the globe, has started to employ non-medical practitioners to extend surgical care capacity (Halter et al 2018). These non-medical practitioners have been integrated into cardiac surgical teams since 1973, the late 1980s and the early 1990s in the United States of America (USA), the United Kingdom (UK) and the KSA, respectively. The role has been defined as follows:

…a non-medical practitioner, working in clinical practice as a member of the extended surgical team, who performs surgical intervention, preoperative and postoperative care under the direction and supervision of a consultant surgeon. (Royal College of Surgeons 2014: p11)

These practitioners are variously referred to as ‘cardiothoracic physician assistants’ (PAs) in the USA, ‘surgical care practitioners’ (SCP) in the UK and ‘surgical assistants’ (SAs) in KSA. In routine practice, they perform conduit harvests such as saphenous vein and/or radial artery harvest in CABG and act as first or second assistants in major cases (Shegafi et al 2020).

In the KSA, where coronary artery disease is one of the leading causes of death, cardiac SAs work in hospitals that differ in their organisational governance, being run either privately or by the Ministry of Health (MOH), the Ministry of Education (MOE) or the Ministry of Defence (MOD) (Almasabi 2013, Mujamammi et al 2020). Currently, there is no available information on the cardiac SA workforce in the KSA, so characteristics such as its demographics and the proportion of KSA to expatriate staff are not known. While cardiothoracic PA and SCP training in the USA and UK involves both hospitals and universities, this is not the case in KSA, where an entirely hospital-based training approach is still undertaken, although little information is available regarding training duration and content (Halter et al 2018).

Much of the available literature on the non-medical workforce in cardiac surgery is anecdotal. A recent systematic review of their impact on clinical outcomes in the UK highlighted a lack of empirical evidence (Shegafi et al 2020). Other concerns have been raised including their degree of role autonomy when seeking informed consent (Nicholas 2018) and associated levels of job satisfaction and staff turnover (Thourani & Miller Jr 2006). To date, no empirical research has attempted to examine these matters in more depth or to investigate ways in which the job might be redesigned to address them. Poor job design, defined as ‘the application of motivational theories to the structure of work for improving productivity and satisfaction’ (Potter 2009: p2), can result in unfavourable outcomes for both organisations and individuals (Parker et al 2019).

### Theoretical framework

The job characteristics model of Hackman and Oldham (1976) has been the standard work design model for academics and practitioners for more than 40 years (Bayona et al 2015). It identifies five job attributes that are linked to employees’ motivation and job satisfaction:

- Skill variety – the degree to which the employee is required to use a wide variety of abilities and skills.
- Task identity – the way in which the worker feels that they have the responsibility for achieving a complete and identifiable task, rather than merely a subsection of it.
- Task significance – the degree to which the job has an effect on others both within and outside the organisation.
- Autonomy – the workers’ degree of independence and self-governing in their work choices and autonomy in their work.
- Feedback from the job – provision of information about performance from the job itself and not from other people.

The job characteristics model was selected to inform the research because of its seminal contribution to the field of work design, emphasis on the connection between job design and job satisfaction, and contemporary relevance. For example, the Work Design Questionnaire (Morgeson & Humphrey 2006) used in this research was informed by the job characteristics model. Hackman and Oldham (1976) suggested that for people to grow in their job role, they require strength, skills, knowledge and a satisfying work context, as these elements moderate the characteristics of the job and work outcomes. Morgeson and Humphrey (2006) have since argued that employees’ job satisfaction can be influenced by task, knowledge, social and contextual characteristics. This study was therefore conducted to determine cardiac SAs’ perspectives toward these job characteristics. It forms part of a larger mixed-methods research project to explore cardiac SAs’ perspectives in the KSA, identify factors which affect job satisfaction, motivation and role autonomy, and consider ways to re-design the job to address these factors.

## Method

### Setting

This study took place in eight cardiac centres in KSA at which cardiac SAs currently work. Two of these centres are in the central region, one in the south, one in the north, two in the western region, and two in the eastern region.

### Sample

The lead researcher developed a sampling frame of all SAs through informal meetings with operating theatre stakeholders in each cardiac centre and by obtaining a list of all SA employees. All cardiac SAs working in clinical roles or in non-clinical areas such as administrative or managerial roles were eligible for inclusion. In total, a potential of 53 cardiac SAs were identified.

### Data collection

An online survey using SurveyMonkey was conducted from July to September 2020 to collect data on the nature of cardiac SAs’ work across KSA. All cardiac SAs were electronically invited to participate in the study, and those who expressed an interest were given information about the study. The study was granted ethical approval from King’s College London (MRSP-19/20-17546) in the UK and King Abdullah Medical City in the KSA (20-705). The researchers conducted the study to respect the participant’s right to withdraw themselves or their data from the study either during or after participation without having to provide a reason. Researchers also ensured that all participants understood that they were under no obligation to take part in a research study. The benefits of participation in the study were outlined in the participant information leaflet (eg gathering information on cardiac SAs job design will help to identify job characteristics that required further redesigning interventions and could improve cardiac SAs job design). Participants were informed that their responses would be anonymous and aggregated so that they would not be identifiable in any published outputs from the study. All survey responses were submitted anonymously, and no identifiable data of any participant was presented. Completing the survey signalled consent to participate.

### Measure

Morgeson and Humphrey’s (2006) Work Design Questionnaire (WDQ) was used with permission. It consists of 77 items relating to four main domains: (1) task characteristics, (2) knowledge characteristics, (3) social characteristics and (4) contextual characteristics. The WDQ uses a Likert scale for participants to indicate the extent they agree with statements about their work characteristics, scoring from 1 = ‘strongly disagree’ to 5 = ‘strongly agree’.

- ***Task characteristics domain:*** This comprises five scales related to both the range of tasks and the nature of the tasks connected with a specific job and how the work is carried out. These scales are ‘autonomy’, ‘task variety’, ‘task significance’, ‘task identity’ and ‘feedback from the job’. Each scale contains four items except for the ‘feedback from the job’ scale (three items) and ‘autonomy’ scale, which is subdivided into three subscales: (a) ‘work scheduling’, (b) ‘decision-making’ and (c) ‘work methods’, with three items in each subscale.
- ***Knowledge characteristics domain:*** This comprises five scales concerning ‘the kinds of knowledge, skill and ability demands that are placed on an individual as a function of what is done on the job’ (Morgeson & Humphrey 2006: p1323). These four-item scales are ‘job complexity’, ‘information processing’, ‘problem-solving’, ‘skill variety’ and ‘specialisation’.
- ***Social characteristic domain*:** This comprises four scales concerning the opportunities that the job offers for social interaction with others. These scales are ‘social support’, ‘interdependence’, ‘interaction outside the organisation’ and ‘feedback from others’. The ‘social support’ scale comprises six items and the ‘interdependence’ scale is divided into two subscales: (a) ‘initiated interdependence’ and (b) ‘received interdependence’, with three items in each. There are four items in the ‘interaction outside the organisation’ scale and three items in the ‘feedback from others’ scale.
- ***Contextual characteristics domain:*** This comprises four scales concerning the context in which work is carried out, including physical conditions and environmental factors. These scales are ‘ergonomics’, ‘physical demands’, ‘work conditions’ and ‘equipment use’. Except for the ‘work conditions’ scale, which comprises six items, all other scales contain three items.

Confirmatory factor analyses performed by the authors indicated support for the factor structure of the WDQ. Subscales demonstrated excellent internal consistency. Cronbach's alpha ranged from 0.64 (ergonomics) to 0.95 (task variety and physical demands), with a mean alpha of 0.86.

### Data analysis

Survey data from the completed questionnaires were coded by the lead author and entered into SPSS version 26. Data were screened for errors using descriptive statistics to check for values falling outside the expected range for each item, thus ensuring that there were no errors in data inputting. Reliability estimates were also performed for each scale. Similar to the original WDQ and other Persian and Spanish versions (Bayona et al 2015, Khandan et al 2018), Cronbach's alpha for the ergonomics scale was 0.59: this may be a result of reverse scoring, as justified by Khandan et al (2018).

High scores indicated that the work design of cardiac SAs was considered good by the respondents, while low scores indicated a need to redesign the cardiac SAs’ job. Even though the WDQ has been translated into seven languages since its publication (Morgeson & Humphrey 2006), there is no benchmark for what is considered a low score. A pragmatic approach was taken towards identifying scales for redesign. A scale was considered for redesign if one or more of the following three criteria were met:

1. The median scale score, having divided by the number of items in the scale, was less than 3 so more respondents disagreed than agreed with the items in the scale.
2. The mean scale score, having divided by the number of items in the scale, had a value less than 3.
3. The scale distribution was multimodal, so it had more than one peak.

Criteria 1 and 2, if met, would typically identify the same scales, but there may be the occasion where they do not. To cover all eventualities, both were used. Hopefully, these three criteria would capture most of the scales that required further review.

## Results

Of the total population of 53 cardiac SAs identified, 66% completed the survey. Table 1 summarises the respondents’ demographics.

**Table 1:** Respondents’ demographic

Demographic		N	%
Gender	Male	25	74.0
	Female	6	18.0
	Prefer not to tell	3	9.0
Age	25–34	21	62
	35–44	10	29.4
	45–54	3	8.8
Qualification	Bachelor’s degree	20	58.8
	Diploma	11	32.4
	Master’s degree	2	5.9
	PhD degree	1	2.9
Experience	≤1 year	1	3.0
	1–5 years	9	26.0
	6–10 years	17	50.0
	≥10 years	7	21.0
Location	Central area	14	41.2
	Eastern area	6	17.7
	Northern area	4	11.8
	Southern area	2	5.9
	Western area	8	23.5

### Task characteristics domain

**Figure 1.**
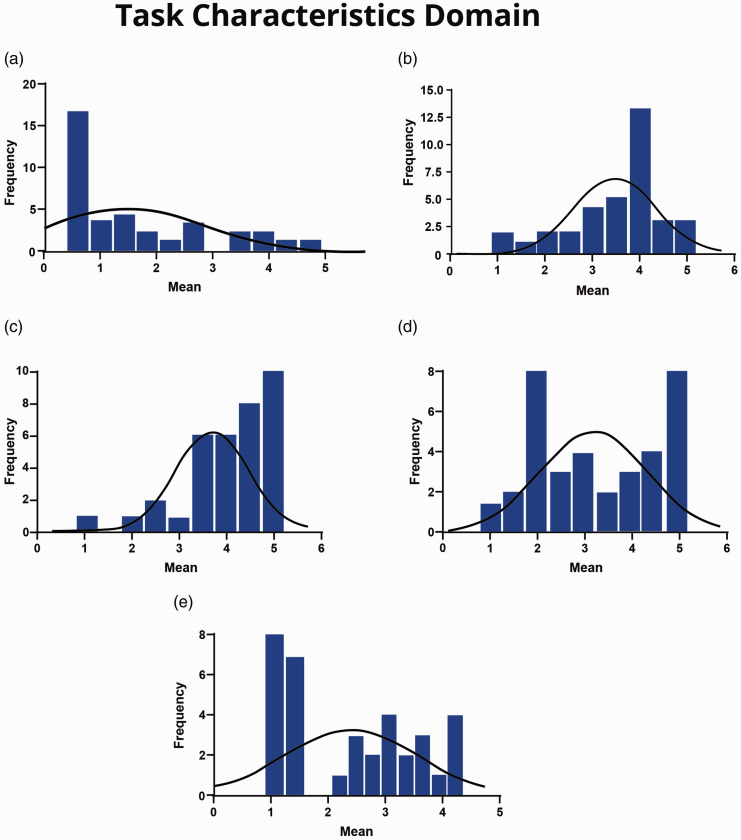
(a) Autonomy scale. (b) Task variety scale. (c) Task significance scale. (d) Task identity scale. (e) Feedback from job scale.

Survey results showed a variation in cardiac SAs’ responses among the scales in this domain. The ‘task variety’ and ‘task significance’ scales were found to be at satisfactory levels, with mean scores of 3.42 and 3.20 (median 3.75 and 3.00, respectively). Additionally, the histogram visualisation for both scales showed that the data were positively skewed (Figure 1(b) and (c)). For the ‘task identity’ scale, although the mean and median scores were at satisfactory levels (3.94 and 4.25, respectively), the histogram visualisation showed a bimodal distribution of respondents’ attitudes toward the identity of their job (Figure 1(d)).

On the other hand, the majority of the cardiac SAs’ responses on the ‘autonomy’ scale showed that all subscales were at an unsatisfactory level. For example, 60% and 23% of cardiac SAs responded with ‘strongly disagree’ and ‘disagree’, respectively, for ‘The job allows me to make my own decisions about how to schedule my work’; 57% and 26% responded with ‘strongly disagree’ and ‘disagree’, respectively, to the statement ‘The job allows me to make a lot of decisions on my own’; 49% and 31% of cardiac SAs responded ‘strongly disagree’ and ‘disagree’, respectively, to the statement ‘The job allows me to decide on my own how to go about doing my work’ (Table 2). Thus, cardiac SAs perceived a lack of autonomy in terms of their work scheduling, decision-making and work methods.

**Table 2:** Task characteristics domain

Domains	Strongly disagree	Disagree	Neither agree nor disagree	Agree	Strongly agree	Total
1- Task characteristics	(n) %	(n) %	(n) %	(n) %	(n) %	(n) %
Autonomy						
Work scheduling autonomy						
Q1. The job allows me to make my own decisions about how to schedule my work.	(21) 60%	(8) 23%	(3) 9%	(3) 9%	(0) 0%	(35) 100%
Q2. The job allows me to decide on the order in which things are done on the job.	(19) 54%	(8) 23%	(3) 9%	(5) 14%	(0) 0%	(35) 100%
Q3. The job allows me to plan how I do my work.	(19) (54%	(7) 20%	(3) 9%	(3) 9%	(3) 9%	(35) 100%
Decision-making autonomy						
Q4. The job gives me a chance to use my personal initiative or judgement in carrying out the work.	(19) 54%	(5) 14%	(5) 14%	(4) 11%	(2) 6%	(35) 100%
Q5. The job allows me to make a lot of decisions on my own.	(20) 57%	(9) 26%	(3) 9%	(2) 6%	(1) 3%	(35) 100%
Q6. The job provides me with significant autonomy in making decisions.	(23) 66%	(4) 11%	(4) 11%	(2) 6%	(2) 6%	(35) 100%
Work methods autonomy						
Q7. The job allows me to make decisions about what methods I use to complete my work.	(18) 51%	(6) 17%	(2) 6%	(5) 14%	(4) 11%	(35) 100%
Q8. The job gives me considerable opportunity for independence and freedom in how I do the work.	(19) 54%	(7) 20%	(2) 6%	(3) 9%	(4) 11%	(35) 100%
Q9. The job allows me to decide on my own how to go about doing my work.	(17) 49%	(11) 31%	(2) 6%	(4) 11%	(1) 3%	(35) 100%
Task variety						
Q10. The job involves a great deal of task variety.	(8) 23%	(5) 14%	(6) 17%	(14) 40%	(2) 6%	(35) 100%
Q11. The job involves doing a number of different things.	(4) 11%	(3) 9%	(4) 11%	(20) 57%	(4) 11%	(35) 100%
Q12. The job requires the performance of a wide range of tasks.	(4) 11%	(4) 11%	(2) 6%	(19) 54%	(6) 17%	(35) 100%
Q13. The job involves performing a variety of tasks.	(3) 9%	(1) 3%	(4) 11%	(21) 60%	(6) 17%	(35) 100%
Task significance						
Q14. The results of my work are likely to significantly affect the lives of other people.	(2) 6%	(5) 14%	(3) 9%	(14) 40%	(11) 31%	(35) 100%
Q15. The job itself is very significant and important in the broader scheme of things.	(1) 3%	(2) 6%	(2) 6%	(12) 34%	(18) 51%	(35) 100%
Q16. The job has a large impact on people outside the organisation.	(1) 3%	(3) 9%	(7) 20%	(10) 29%	(14) 40%	(35) 100%
Q17. The work performed on the job has a significant impact on people outside the organisation.	(1) 3%	(4) 11%	(9) 26%	(9) 26%	(12) 34%	(35) 100%
Task identity						
Q18. The job involves completing a piece of work that has an obvious beginning and end.	(4) 11%	(8) 23%	(5) 14%	(10) 29%	(8) 23%	(35) 100%
Q19. The job is arranged so that I can do an entire piece of work from beginning to end.	(4) 11%	(10) 29%	(5) 14%	(7) 20%	(9) 26%	(35) 100%
Q20. The job provides me the chance to completely finish the pieces of work I begin.	(5) 14%	(11) 31%	(4) 11%	(7) 20%	(8) 23%	(35) 100%
Q21. The job allows me to complete work I start.	(2) 6%	(11) 31%	(6) 17%	(8) 23%	(8) 23%	(35) 100%
Feedback from job						
Q22. The work activities themselves provide direct and clear information about the effectiveness (eg quality and quantity) of my job performance.	(10) 29%	(9) 26%	(6) 17%	(9) 26%	(1) 3%	(35) 100%
Q23. The job itself provides feedback on my performance.	(14) 40%	(3) 9%	(6) 17%	(7) 20%	(5) 14%	(35) 100%
Q24. The job itself provides me with information about my performance.	(16) 46%	(1) 3%	(5) 14%	(8) 23%	(5) 14%	(35) 100%

As illustrated in the histogram, the data on the autonomy scale are negatively skewed (Figure 1(a)), with a mean of 1.89 and median of 1.44 (Table 6). Moreover, data for the ‘feedback from job’ scale were at an unsatisfactory level. For example, 46% and 3% of the cardiac SAs responded with ‘strongly disagree’ and ‘disagree’, respectively, to the statement ‘The job itself provides me with information about my performance’ (mean = 2.55, median = 2.67). However, in agreement with the ‘task identity’ scale, the histogram visualisation showed a bimodal distribution of cardiac SAs’ attitudes (Figure 1(e)). The cardiac SAs’ responses to each item in the ‘task characteristics’ domain scales are summarised in Table 2.

### Knowledge characteristics domain

**Figure 2.**
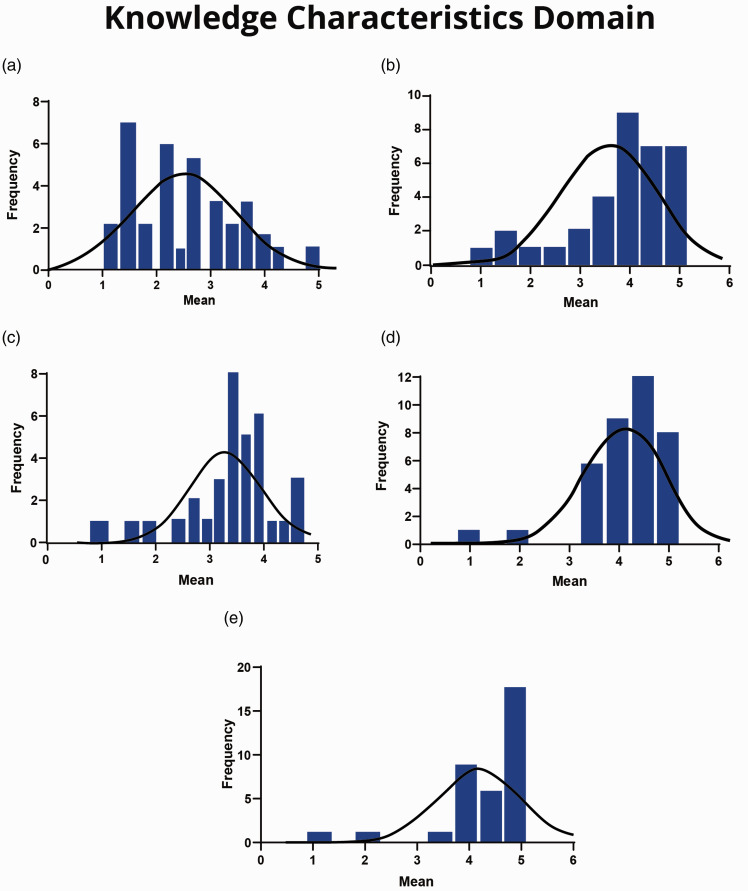
(a) Job complexity scale. (b) Information processing scale. (c) Problem solving scale. (d) Skill variety scale. (e) Specialisation scale.

All of the scales in this domain were found to be at satisfactory levels except for the ‘job complexity’ scale (mean = 2.32, median = 2.13). For example, on the statement ‘The job involves performing relatively simple tasks’, 18% and 44% of the respondents ‘agree’ and ‘strongly agree’, respectively (Table 3). The overall mean and median of cardiac SAs’ attitudes to information processing, problem-solving, skill variety and specialisation scales are presented in Table 6. Additionally, Figure 2(a) to (e) illustrates the distribution of the data for all scales in this domain and Table 3 summarises the cardiac SAs’ responses for each item in the ‘knowledge characteristics’ domain.

**Table 3:** Knowledge characteristics domain

Domains	Strongly disagree	Disagree	Neither agree nor disagree	Agree	Strongly agree	Total
2- Knowledge characteristics	(n) %	(n) %	(n) %	(n) %	(n) %	(n) %
Job complexity						
Q25. The job requires that I only do one task or activity at a time.	(2) 6%	(7) 21%	(7) 21%	(15) 44%	(3) 9%	(34) 100%
Q26. The tasks on the job are simple and uncomplicated.	(1) 3%	(2) 6%	(8) 24%	(10) 29%	(13) 38%	(34) 100%
Q27. The job comprises relatively uncomplicated tasks.	(1) 3%	(6) 18%	(7) 21%	(9) 26%	(11) 32%	(34) 100%
Q28. The job involves performing relatively simple tasks.	(1) 3%	(7) 21%	(5) 15%	(6) 18%	(15) 44%	(34) 100%
Information processing						
Q29. The job requires me to monitor a great deal of information.	(4) 12%	(2) 6%	(3) 9%	(17) 50%	(8) 24%	(34) 100%
Q30. The job requires that I engage in a large amount of thinking.	(4) 12%	(3) 9%	(1) 3%	(16) 47%	(10) 29%	(34) 100%
Q31. The job requires me to keep track of more than one thing at a time.	(1) 3%	(4) 12%	(6) 18%	(10) 29%	(13) 38%	(34) 100%
Q32. The job requires me to analyse a lot of information.	(3) 9%	(3) 9%	(3) 9%	(13) 38%	(12) 35%	(34) 100%
Problem solving						
Q33. The job involves solving problems that have no obvious correct answer.	(3) 9%	(2) 6%	(9) 26%	(18) 53%	(2) 6%	(34) 100%
Q34. The job requires me to be creative.	(2) 6%	(4) 12%	(6) 18%	(16) 47%	(6) 18%	(34) 100%
Q35. The job often involves dealing with problems that I have not met before.	(2) 6%	(2) 6%	(7) 21%	(21) 62%	(2) 6%	(34) 100%
Q36. The job requires unique ideas or solutions to problems.	(1) 3%	(4) 12%	(12) 35%	(12) 35%	(5) 15%	(34) 100%
Skill variety						
Q37. The job requires a variety of skills.	(1) 3%	(1) 3%	(2) 6%	(19) 56%	(11) 32%	(34) 100%
Q38. The job requires me to utilise a variety of different skills in order to complete the work.	(1) 3%	(2) 6%	(1) 3%	(15) 44%	(15) 44%	(34) 100%
Q39. The job requires me to use a number of complex or high-level skills.	(1) 3%	(1) 3%	(5) 15%	(16) 47%	(11) 32%	(34) 100%
Q40. The job requires the use of a number of skills.	(1) 3%	(2) 6%	(4) 12%	(10) 29%	(17) 50%	(34) 100%
Specialisation						
Q41. The job is highly specialised in terms of purpose, tasks, or activities.	(1) 3%	(1) 3%	(3) 9%	(12) 35%	(17) 50%	(34) 100%
Q42. The tools, procedures, materials, and so forth used on this job are highly specialised in terms of purpose.	(1) 3%	(1) 3%	(3) 9%	(10) 29%	(19) 56%	(34) 100%
Q43. The job requires very specialised knowledge and skills.	(1) 3%	(1) 3%	(0) 0%	(13) 38%	(19) 56%	(34) 100%
Q44. The job requires a depth of knowledge and expertise.	(1) 3%	(1) 3%	(2) 6%	(8) 24%	(22) 65%	(34) 100%

### Social characteristics domain

**Figure 3.**
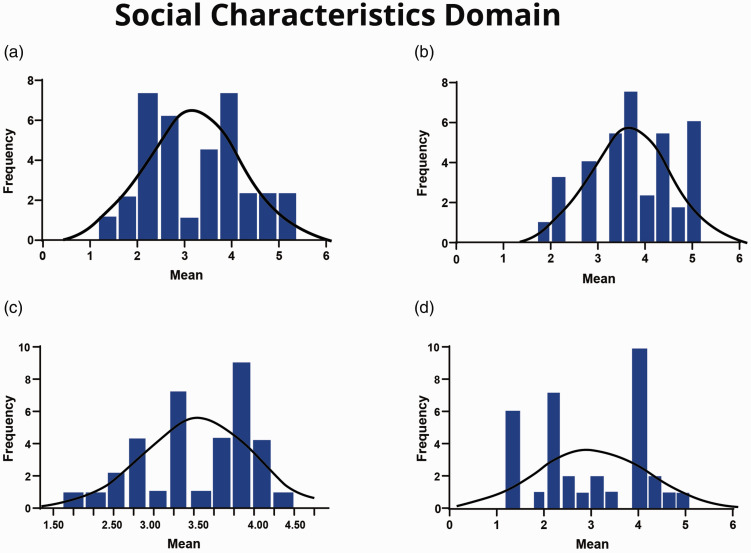
(a) Social support scale. (b) Interdependence scale. (c) Interaction outside organisation scale. (d) Feedback from others scale.

Two of the four scales that comprised the ‘social characteristics’ domain were found to be at unsatisfactory levels: on the ‘feedback from others’ scale (mean = 2.84, median = 2.83), 18% and 26% responded with ‘strongly disagree’ and ‘disagree’, respectively, to the statement ‘I receive a great deal of information from my manager and co-workers about my job performance’ (Table 4). Figure 3(d) illustrates the distribution of responses on this scale. Additionally, although the mean and median of cardiac SAs’ scores on the ‘social support’ scale were 3.40 and 3.42, the distribution of data was bimodal, as illustrated in Figure 3(a). In contrast, both the ‘interdependence’ (mean = 3.80, median = 3.92) and ‘interaction outside organisation’ (mean = 3.23, median = 3.38) scales were found to be at satisfactory levels.

**Table 4:** Social characteristics domain

Domains	Strongly disagree	Disagree	Neither agree nor disagree	Agree	Strongly agree	Total
3- Social characteristics	(n) %	(n) %	(n) %	(n) %	(n) %	(n) %
Social support						
Q45. I have the opportunity to develop close friendships in my job.	(1) 3%	(8) 24%	(7) 21%	(12) 35%	(6) 18%	(34) 100%
Q46. I have the chance in my job to get to know other people.	(0) 0%	(2) 6%	(5) 15%	(22) 65%	(5) 15%	(34) 100%
Q47. I have the opportunity to meet with others in my work.	(0) 0%	(4) 12%	(12) 35%	(13) 38%	(5) 15%	(34) 100%
Q48. My supervisor is concerned about the welfare of the people that work for him/her.	(1) 3%	(13) 38%	(9) 26%	(7) 21%	(4) 12%	(34) 100%
Q49. People I work with take a personal interest in me.	(4) 12%	(11) 32%	(8) 24%	(8) 24%	(3) 9%	(34) 100%
Q50. People I work with are friendly.	(0) 0%	(1) 3%	(14) 41%	(14) 41%	(5) 15%	(34) 100%
Interdependence						
Initiated interdependence						
Q51. The job requires me to accomplish my job before others complete their job.	(2) 6%	(6) 18%	(4) 12%	(12) 35%	(10) 29%	(34) 100%
Q52. Other jobs depend directly on my job.	(1) 3%	(5) 15%	(5) 15%	(11) 32%	(12) 35%	(34) 100%
Q53. Unless my job gets done, other jobs cannot be completed.	(1) 3%	(4) 12%	(10) 29%	(6) 18%	(13) 38%	(34) 100%
Received interdependence						
Q54. The job activities are greatly affected by the work of other people.	(2) 6%	(2) 6%	(5) 15%	(16) 47%	(9) 26%	(34) 100%
Q55. The job depends on the work of many different people for its completion.	(0) 0%	(4) 12%	(4) 12%	(17) 50%	(9) 26%	(34) 100%
Q56. My job cannot be done unless others do their work.	(1) 3%	(3) 9%	(8) 24%	(11) 32%	(11) 32%	(34) 100%
Interaction outside organisation						
Q57. The job requires spending a great deal of time with people outside my organisation.	(1) 3%	(10) 29%	(10) 29%	(11) 32%	(2) 6%	(34) 100%
Q58. The job involves interaction with people who are not members of my organisation.	(2) 6%	(6) 18%	(6) 18%	(19) 56%	(1) 3%	(34) 100%
Q59. On the job, I frequently communicate with people who do not work for the same organisation as I do.	(1) 3%	(5) 15%	(12) 35%	(15) 44%	(1) 3%	(34) 100%
Q60. The job involves a great deal of interaction with people outside my organisation.	(0) 0%	(9) 26%	(11) 32%	(12) 35%	(2) 6%	34 100%
Feedback from others						
Q61. I receive a great deal of information from my manager and coworkers about my job performance.	(6) 18%	(9) 26%	(4) 12%	(10) 29%	(5) 15%	()34 100%
Q62. Other people in the organisation, such as managers and coworkers, provide information about the effectiveness (eg quality and quantity) of my job performance.	(7) 21%	(11) 32%	(3) 9%	(12) 35%	(1) 3%	34 100%
Q63. I receive feedback on my performance from other people in my organisation (such as my manager or coworkers).	(7) 21%	(9) 26%	(4) 12%	(9) 26%	(5) 15%	34 100%

Figure 3(b) and (c) illustrates the distributions of these two scales. Table 4 summarises cardiac SAs’ responses for each item in the ‘social characteristics’ domain.

### Work characteristics context domain

There were variations in the respondents’ attitudes toward the scales in the ‘work characteristics’ context.

**Figure 4.**
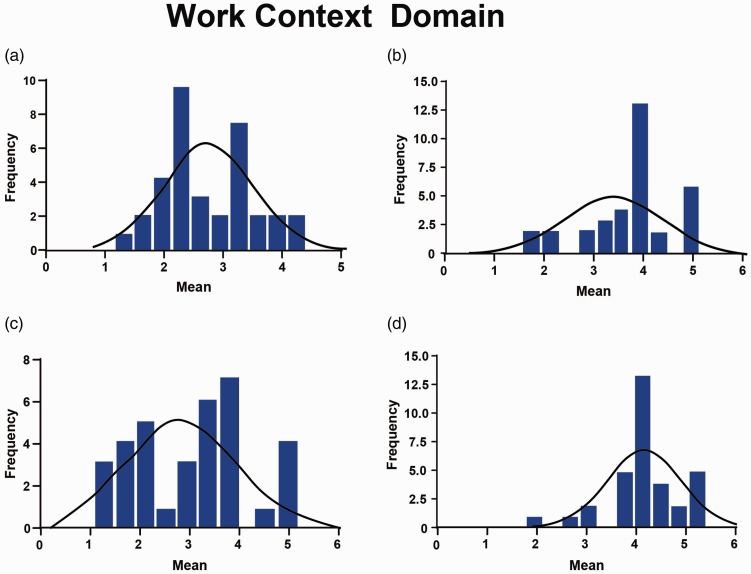
(a) Ergonomics scale. (b) Physical demands scale. (c) Work conditions scale. (d) Equipment use scale.

The ‘ergonomics’ and ‘work conditions’ scales were found to be at unsatisfactory levels. For the ‘ergonomics’ scale (mean = 2.79, median = 2.67), 9% and 44% of the respondents ‘strongly disagreed’ and ‘disagreed’, respectively, with the statement ‘The seating arrangements on the job are adequate (eg ample opportunities to sit, comfortable chairs, good postural support)’ (Table 5). In addition, as illustrated in Figure 4(a) and (c), the distribution of responses for this scale was bimodal.

**Table 5:** Work context domain

Domains	Strongly disagree		Disagree	Neither agree nor disagree	Agree	Strongly agree	Total
4-Work context	(n) %		(n) %	(n) %	(n) %	(n) %	(n) %
Ergonomics							
Q64. The seating arrangements on the job are adequate (eg ample opportunities to sit, comfortable chairs, good postural support).	(3) 9%	(15) 44%		(6) 18%	(9) 26%	(1) 3%	(34) 100%
Q65. The workplace allows for all size differences between people in terms of clearance, reach, eye height, leg room, etc.	(4) 12%	(13) 38%		(7) 21%	(8) 24%	(2) 6%	(34) 100%
Q66. The job involves excessive reaching.	(3) 9%	(7) 21%		(14) 41%	(9) 26%	(1) 3%	(34) 100%
Physical demands							
Q67. The job requires a great deal of muscular endurance.	(0) 0%	(4) 12%		(12) 35%	(11) 32%	(7) 21%	(34) 100%
Q68. The job requires a great deal of muscular strength.	(2) 6%	(3) 9%		(9) 26%	(14) 41%	(6) 18%	(34) 100%
Q69. The job requires a lot of physical effort.	(0) 0%	(5) 15%		(2) 6%	(9) 26%	(18) 53%	(34) 100%
Work conditions							
Q70. The workplace is free from excessive noise.	(12) 35%	(3) 9%		(6) 18%	(8) 24%	(5) 15%	(34) 100%
Q71. The climate at the workplace is comfortable in terms of temperature and humidity.	(2) 6%	(7) 21%		(6) 18%	(13) 38%	(6) 18%	(34) 100%
Q72. The job has a low risk of accident.	(12) 35%	(9) 26%		(4) 12%	(7) 21%	(2) 6%	(34) 100%
Q73. The job takes place in an environment free from health hazards (eg chemicals, fumes, etc).	(12) 35%	(6) 18%		(5) 15%	(6) 18%	(5) 15%	(34) 100%
Q74. The job occurs in a clean environment.	(1) 3%	(5) 15%		(3) 9%	(13) 38%	(12) 35%	(34) 100%
Equipment use							
Q75. The job involves the use of a variety of different equipment.	(0) 0%	(1) 3%		(1) 3%	(20) 59%	(12) 35%	(34) 100%
Q76. The job involves the use of complex equipment or technology.	(0) 0%	(2) 6%		(3) 9%	(20) 59%	(9) 26%	(34) 100%
Q77. A lot of time was required to learn the equipment used on the job.	(2) 6%	(1) 3%		(7) 21%	(18) 53%	(6) 18%	(34) 100%

Similarly, for the ‘work conditions’ scale, the mean and median were 2.99 and 3.20, respectively, and as illustrated in Table 6, the distribution of responses was bimodal. On the other hand, cardiac SAs indicated satisfaction on the ‘physical demands’ (mean = 3.74, median= 4.00) and ‘equipment uses’ (mean = 4.02, median = 4.00) scales. Figure 4(b) and (d) illustrates the scale distributions. Table 5 summarises cardiac SAs’ responses for each item in the ‘work characteristics’ domain.

**Table 6:** Scales descriptive statistics

Work design questionnaire		Autonomy scale	Task variety scale	Task significance scale	Task identity scale	Feedback from job scale	Job complexity scale	Information processing scale	Problem solving scale	Skill variety scale	Specialisation scale	Social support scale	Interdependence scale	Interaction outside organisation scale	Feedback from others scale	Ergonomics scale	Physical demands scale	Work conditions	Equipment use scale
N	Valid	35	35	35	35	35	34	34	34	34	34	34	34	34	34	34	34	34	34
	Missing	0	0	0	0	0	1	1	1	1	1	1	1	1	1	1	1	1	1
Mean		1.89	3.42	3.20	3.94	2.55	2.32	3.78	3.51	4.13	4.36	3.40	3.80	3.23	2.84	2.79	3.78	2.99	4.02
Median		1.44	3.75	3.00	4.25	2.67	2.13	4.00	3.50	4.25	4.75	3.42	3.92	3.38	2.83	2.67	4.00	3.20	4.00
Standard deviation		1.07	1.04	1.29	0.97	1.36	0.98	1.07	0.82	0.82	0.88	0.79	0.93	0.66	1.27	0.79	0.91	1.08	0.67
Minimum		1.00	1.00	1.00	1.00	1.00	1.00	1.00	1.00	1.00	1.00	2.00	1.83	1.75	1.00	1.33	1.67	1.40	2.00
Maximum		4.33	5.00	5.00	5.00	4.67	4.75	5.00	4.75	5.00	5.00	5.00	5.00	4.25	5.00	4.33	5.00	5.00	5.00

## Discussion

This cross-sectional survey highlights the perspectives of cardiac SAs in KSA about their work environment and job role. This study found that ‘autonomy’, ‘task identity’ and ‘feedback from the job’ scales (‘task characteristics’ domain), ‘job complexity’ (‘knowledge characteristics’ domain), ‘feedback from others’ and ‘social support’ scales (‘social characteristics’ domain) and the **‘**ergonomics’ and ‘work conditions’ scales (‘work characteristics’ domain) were all rated as unsatisfactory, indicating what aspects a job redesign should prioritise in order to address these areas of concerns.

Characteristics such as autonomy and task identity were among factors raised by Hix and Fernandes (2020) in their qualitative research of PAs in Germany. The authors concluded that currently, German PAs are not permitted to diagnose, create treatment plans, manage anaesthesia, or begin therapies; they can only ‘participate’ in these clinical activities under physicians’ direction. However, Hix and Fernandes (2020) did not include cardiac PAs in their study.

Interestingly, more than 60% of cardiac SAs perceived that they are doing simple tasks and that their job is not challenging enough. According to DePalma et al (2019), who conducted a study of job satisfaction in cardiovascular medicine PAs in the US, most who responded (87.3%) were satisfied or very satisfied with their jobs. They related their high job satisfaction to job factors related to challenge and high levels of autonomy. However, the DePalma et al (2019) study had several limitations, including the use of a non-valid instrument and small sample size.

To the best of our knowledge, this opinion survey is the first to be conducted focusing exclusively on non-medical practitioners in the cardiac surgical field. This claim is supported by the recent published research on PAs job satisfaction by Hooker et al (2015) and Hoff et al (2019) who conducted narrative and systematic reviews respectively to examine the empirical evidence on PAs in all specialties. However, Hoff et al (2019) added evidence on nurse practitioners’ job satisfaction and neither of the reviews included any study on cardiac PAs. Both reviews concluded that research on PA job satisfaction is underdeveloped, inadequate and outdated. Thus, the knowledge on PA job satisfaction in general is limited.

The results of this study can be used to prioritise the aspects of the cardiac SA role which need to be redesigned in order to improve job satisfaction and related individual and organisational outcomes such as recruitment, career progression and retention in the local context of the KSA. While this study has been conducted in one country and only focused on one non-medical practitioner role, the findings may be of interest and resonate with both cardiac surgical assistants working in other countries and also other non-medical practitioners working in different fields. It is recommended to replicate the survey in different settings and in different non-medical practitioner samples to determine the extent to which these findings are context and role dependent. Importantly, where cardiac non-medical assistants undertake postgraduate and accredited study, results on satisfaction and perceived job characteristics may be very different indeed. Such an analysis could yield important conclusions both for countries such as the UK and US, and for other countries seeking to progress the agenda on non-medical workforce design.

Due to its descriptive nature, this research does not provide a detailed explanation of the reasons why cardiac SAs perceived some characteristics to be at a non-satisfactory level. Therefore, the next stage will be undertaken qualitatively to explore how cardiac SAs perceive their job in relation to key motivational, social and contextual characteristics, and what can be done to improve their job design. However, since this research is limited to the KSA context, the results might not be applicable to other non-medical practitioners who work as part of extended cardiac surgical teams in other countries. Therefore, further organisational psychology research needs to be undertaken on non-medical practitioners’ status in other contexts.

## Conclusion

This research provides insight into aspects of cardiac SAs’ role characteristics and contributes to the body of knowledge about organisational psychology of non-medical practitioners in the cardiac surgical field. Overall, all WDQ scales were shown to be at satisfactory level except for the ‘autonomy’, ‘task identity’, ‘feedback from the job’, ‘job complexity’, ‘social support’, ‘feedback from others’, ‘ergonomic’ and ‘work condition’ scales, which were found to be rated lower than the threshold by cardiac SAs. These results may guide policy makers, health administrators and employers to create more welcoming professional environments for cardiac SAs. Given the growth of cardiothoracic operations, the role of the surgical care assistant needs to be further developed to address the job design issues raised.

*No competing interests declared*
